# Supplementary material for: Mechanisms governing the pioneering and redistribution capabilities of the non-classical pioneer PU.1
Source: Nat Commun. 2020 Jan 21;11:402. doi: 10.1038/s41467-019-13960-2 (PMC6972792; doi:10.1038/s41467-019-13960-2)
Supplement: Supplementary file 7 — Source data [file 41467_2019_13960_MOESM7_ESM.zip › Source_Data/FigureS2/FigureS2B_MotifScanOutput/KmeansCluster4/homerResults/motif8.similar.html]

motif8

## Information for motif8

A
G
C
T
A
C
G
T
C
G
A
T
C
T
A
G
T
G
C
A
C
T
A
G
A
T
C
G
C
G
T
A
G
T
C
A
A
T
C
G
  
Reverse Opposite:  

A
T
G
C
A
C
G
T
C
G
A
T
A
T
G
C
A
G
T
C
A
C
G
T
G
A
T
C
C
G
T
A
T
G
C
A
C
T
G
A
  

|  |  |
| --- | --- |
| p-value: | 1e-118 |
| log p-value: | -2.727e+02 |
| Information Content per bp: | 1.751 |
| Number of Target Sequences with motif | 470.0 |
| Percentage of Target Sequences with motif | 10.41% |
| Number of Background Sequences with motif | 1072.7 |
| Percentage of Background Sequences with motif | 2.57% |
| Average Position of motif in Targets | 102.3 +/- 48.0bp |
| Average Position of motif in Background | 100.4 +/- 74.5bp |
| Strand Bias (log2 ratio + to - strand density) | 0.3 |
| Multiplicity (# of sites on avg that occur together) | 1.03 |
| Motif File: | file (matrix) reverse opposite |

### Similar de novo motifs found

|  |  |  |  |  |  |  |  |
| --- | --- | --- | --- | --- | --- | --- | --- |
| Rank | Match Score | Redundant Motif | P-value | log P-value | % of Targets | % of Background | Motif file |
| 1 | 0.773 | A T C G C A G T G C A T T A G C A G T C G C A T T G A C T G C A G T C A C G A T | 1e-108 | -248.716338 | 8.42% | 1.83% | motif file (matrix) |
| 2 | 0.799 | G C A T G C A T G A C T A C G T C A G T A T C G T G C A C T A G C A T G T G C A C T G A G T A C G A T C | 1e-32 | -74.586796 | 1.37% | 0.12% | motif file (matrix) |
